# 2-{[(Biphenyl-2-yl)diazen­yl]methyl­idene}-1,3,3-trimethyl­indoline

**DOI:** 10.1107/S1600536811008890

**Published:** 2011-03-15

**Authors:** Graeme J. Gainsford, Mohamed Ashraf, Andrew J. Kay

**Affiliations:** a Industrial Research Limited, PO Box 31-310, Lower Hutt, New Zealand

## Abstract

The title mol­ecule, C_24_H_23_N_3_, shows some delocalization of charge based on the small [8.0 (2)°] angle between the indolin-2-yl­idene ring system and the link methyl­diazene C_2_N_2_ atom plane. A further twist of 17.2 (3)° is subtended between the C_2_N_2_ plane and its attached benzene ring. The dihedral angle between the biphenyl rings is 47.96(14)°. In the crystal, the mol­ecules pack *via* C—H⋯π attractive inter­actions.

## Related literature

For applications of azo compounds, see: Möhlmann & van der Vorst (1989 ▶); Zollinger (1987 ▶). For related compounds, see Jones (2004 ▶); Jones & Chrapkowski (2004 ▶); Gainsford *et al.* (2008 ▶). For a description of the Cambridge Structural Database, see: Allen (2002 ▶).
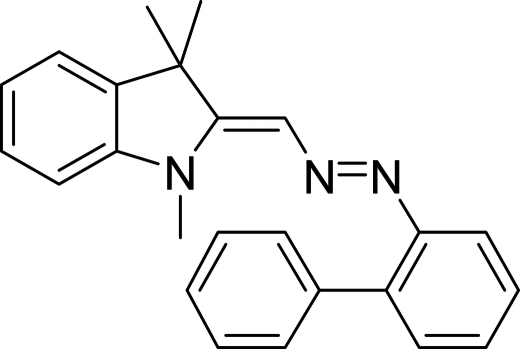


## Experimental

### 

#### Crystal data

C_24_H_23_N_3_
*M* *_r_* = 353.45
Orthorhombic, 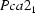

*a* = 14.2968 (17) Å
*b* = 8.2407 (10) Å
*c* = 16.2671 (17) Å
*V* = 1916.5 (4) Å^3^
*Z* = 4
Mo *K*α radiation
μ = 0.07 mm^−1^
*T* = 116 K
0.70 × 0.20 × 0.10 mm

#### Data collection

Bruker APEXII CCD diffractometer
Absorption correction: multi-scan [Blessing (1995 ▶) and *SADABS* (Bruker, 2005 ▶)] *T*_min_ = 0.640, *T*_max_ = 0.746
34258 measured reflections
1783 independent reflections
1764 reflections with *I* > 2σ(*I*)
*R*_int_ = 0.041

#### Refinement

*R*[*F* ^2^ > 2σ(*F* ^2^)] = 0.037
*wR*(*F* ^2^) = 0.086
*S* = 1.23
1783 reflections
247 parameters
1 restraint
H-atom parameters constrained
Δρ_max_ = 0.19 e Å^−3^
Δρ_min_ = −0.16 e Å^−3^

### 

Data collection: *APEX2* (Bruker, 2005 ▶); cell refinement: *SAINT* (Bruker, 2005 ▶); data reduction: *SAINT*; program(s) used to solve structure: *SHELXS97* (Sheldrick, 2008 ▶); program(s) used to refine structure: *SHELXL97* (Sheldrick, 2008 ▶); molecular graphics: *ORTEP-3* (Farrugia, 1997 ▶) and *Mercury* (Macrae *et al.*, 2008 ▶); software used to prepare material for publication: *SHELXL97* and *PLATON* (Spek, 2009 ▶).

## Supplementary Material

Crystal structure: contains datablocks global, I. DOI: 10.1107/S1600536811008890/ez2238sup1.cif

Structure factors: contains datablocks I. DOI: 10.1107/S1600536811008890/ez2238Isup2.hkl

Additional supplementary materials:  crystallographic information; 3D view; checkCIF report

## Figures and Tables

**Table 1:** Hydrogen-bond geometry (Å, °) *Cg*1, *Cg*2 and *Cg*3 are the centroids of the C19–C24, C4–C9 and C13–C18 rings, respectively.

*D*—H⋯*A*	*D*—H	H⋯*A*	*D*⋯*A*	*D*—H⋯*A*
C1—H1*C*⋯*Cg*1^i^	0.98	2.86	3.582 (3)	131
C11—H11*B*⋯*Cg*3^ii^	0.98	2.74	3.721 (3)	179
C22—H22⋯*Cg*2^iii^	0.95	2.76	3.645 (3)	155

Symmetry codes: (i) 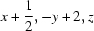; (ii) 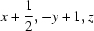; (iii) 
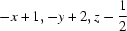
.

**Fig. 1.**
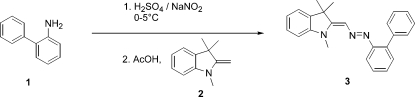
Reaction scheme showing the synthetic procedure for obtaining the title compound.

**Fig. 2.**
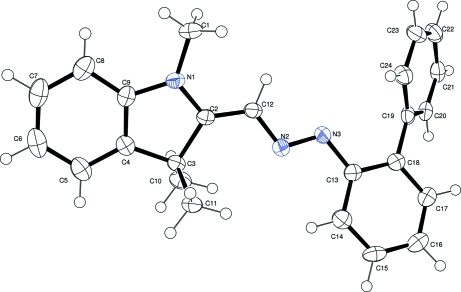
Molecular structure of the asymmetric unit (Farrugia, 1997); displacement ellipsoids are shown at the 50% probability level. H atoms not shown for clarity.

**Fig. 3.**
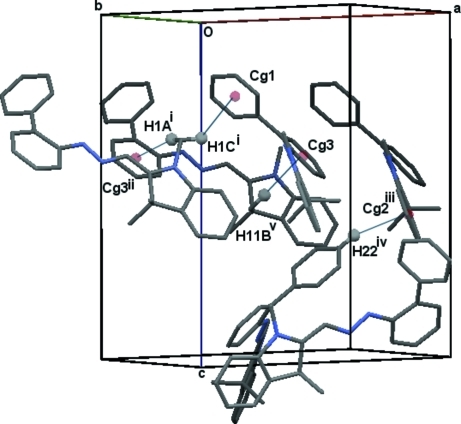
Partial packing diagram of the unit cell showing key interactions (see text and Table 1) [Macrae et al., 2008]. Only significant H atoms are shown as balls for clarity. Symmetry (i) x - 1/2, 2 - y, z (ii) 1/2 - x, 1 - y, z (iii) x, y - 1, z (iv) 1 - x, 1 - y, 1/2 + z (v) x - 1/2, 1 - y, z. Ring centres shown as red balls are Cg1 (C19–C24), Cg2 (C4–C9) and Cg3 (C13–C18).

## supplementary crystallographic information

### Comment

Azo compounds are integral to a number of fields of organic chemistry. While they played a key part in the development of the dye industry (Zollinger, 1987) they are also finding application as the enabling materials for a range of fields including nonlinear optics, photochromism and holographic recording. Dyes based on azobenzene typically exhibit high extinction coefficients and their absorption maxima can usually be tailored to lie anywhere in the visible region *via* appropriate substitution onto the phenyl rings. Furthermore due to their geometrically rigid structures and large aspect ratios, azobenzene compounds are ideal as mesogens (Möhlmann & van der Vorst, 1989).

Recently, we have become interested in developing photoswitchable molecules in order to alter the refractive index of a given material *via* photo-induced rather than electrically induced means (as occurs in nonlinear optical materials). As part of this we have been exploring how the placement of different substituents (*e.g.* donors, acceptors or neutral) on the backbone of various azo dyes affects the speed and reversibility of the photo- and thermal isomerization processes. Included in these studies have been a suite of compounds containing an indoline donor, an azo linker and a variety of substituents attached to the terminal nitrogen atom of the azo moiety. The molecules are easily prepared *via* diazotization of the corresponding aryl amine of the terminal substituent and coupling of the resultant diazonium salt with Fisher's base. The reaction is exemplified in Fig. 1 using 2-aminobiphenyl, 1, as the aromatic amine and which yielded the title compound, 3, in 80% yield.

Compound REFCODES below are from the Cambridge Structural Database (Version 5.31, with Aug 2010 updates; Allen, 2002). The asymmetric unit contents and labels of 3 are shown in Fig. 2. The bond lengths and angles, with the exception of those involving atom C12, are essentially identical with those reported by Jones in 2004 [EZUCUX, EZUDAE (Jones, 2004)) and YADTIH (Jones & Chrapkowski (2004)]. In these latter three compounds, where only a phenyl (or *para*-substituted phenyl) ring was bound to N3, an additional indol-2-ylidene ring was bonded to C12 rather than the hydrogen here (H12). The mean C12—C2 and C12—N2 distances for the three structures were 1.386 (5) and 1.395 (2) Å compared with 1.361 (4) and 1.362 (4) Å here. There are smaller consistent, but barely significant, differences with the N1—C9 and N1—C2 average bond lengths being longer (0.11 (5) Å) and shorter (0.08 (5) °) than found here. These differences can be attributed to extended charge delocalization through the N1—C2—C12—N2 chain that is not observed in the three reference cationic structures.

There is minor buckling beteween the 5- & 6-membered rings in the indol-2-ylidene ring of 1.09 (14) ° compared with 0.46 (14) ° in EZUDAE. The interplanar angles confirm the consistent twist along the delocalization plane: 8.0 (2) ° between the indol-2-ylidene (N1, C2–C9) and the C2,C12,N2,N3 planes, and a further 17.2 (3) ° angle subtended between the latter and the phenyl ring (C14–C18). The biphenyl rings are at 47.96 (14) ° to each other. This is another point of difference with the related compounds, where the "out of plane" indol-2-ylidene ring bound to C12 is at ~80 ° to the mean plane through the phenyl ring bound to N3.

The molecules are held in the lattice by a concerted set of C–H···π interactions shown in Table 1 and Fig. 3. Entry 2 in Table 1 is included because the second methyl hydrogen on C1 (H1A) interacts with atoms C14 & C15 in an adjacent (*Cg*3) ring. We note that the acidic proton H12 is not positioned to interact with adjacent N2 or N3 acceptors as observed in related compounds with cyano N atoms (*e.g.* structure (II) in Gainsford *et al.*, 2008). This packing highlights the main difference between this structure and the reference compounds which have extensive hydrogen bonding (C–H···O) to the perchlorate anion and no significant C–H···π interactions.

### Experimental

To conc. sulfuric acid (4 ml) was added 2-aminobiphenyl 1 (5 mmol) and the reaction was stirred and cooled to 273–278 K. A solution of sodium nitrite (380 mg, 5.5 mol) in 10 ml of water was added slowly and the reaction stirred at 273–278 K for 30 min. To this mixture, was added a solution of Fisher's base, 2, (865 mg, 5 mmol) in 20 ml of glacial acetic acid and the reaction was then stirred for a further 2 h at 273–278 K. The reaction mixture was then poured into water and neutralized with aqueous sodium carbonate. The resulting precipitate was collected by filtration, washed with water and dried over sodium sulfate. Recrystallization (ethanol) gave the title compound 3 as a deep red solid; Yield: 80%. Crystals were prepared by slow evaporation in methanol. m.p. 417.8–419.3 K. ^1^H NMR (DMSO-d_6_, 500 MHz): 7.58 (d, 1H, *J* = 10 Hz), 7.46–7.40 (m, 5H), 7.35–7.31 (m, 2H), 7.29–7.25 (m, 2H), 7.22 (s, 1H), 7.09 (d, 1H, *J* = 10 Hz), 7.02 (t, 2H), 3.31(s, 3H), 1.71(s, 6H). ^13^C NMR (DMSO-d_6_, 125 MHz): 28.66, 29.58, 108.23, 115.62, 121.61, 121.87, 126.58, 126.99, 127.54, 127.81, 128.05, 130.30, 131.53, 137.72, 139.35, 139.70, 144.08, 150.79, 165.97. Mass spec: found: *M*^+^ 354.1972; 354.1970 (calc); Δ = 0.6 p.p.m..

### Refinement

In the absence of significant anomalous scattering, the values of the Flack parameter were indeterminate. Accordingly, the Friedel-equivalent reflections were merged prior to the final refinements. Two reflections affected by the backstop were omitted from the refinements (using *OMIT*) and three others were deemed to be outliers. The methyl H atoms were constrained to an ideal geometry (C—H = 0.98 Å) with *U*_iso_(H) = 1.5*U*_eq_(C), but were allowed to rotate freely about the adjacent C—C bond. All other H atoms were placed in geometrically idealized positions and constrained to ride on their parent atoms with C—H distances of 1.00 (primary), 0.99 (methylene) or 0.95 (phenyl) Å with *U*_iso_(H) = 1.2*U*_eq_(C).

### Crystal data

**Table:**

C_24_H_23_N_3_	*F*(000) = 752
*M**_r_* = 353.45	*D*_x_ = 1.225 Mg m^−^^3^
Orthorhombic, *P**c**a*2_1_	Mo *K*α radiation, λ = 0.71073 Å
Hall symbol: P 2c -2ac	Cell parameters from 9914 reflections
*a* = 14.2968 (17) Å	θ = 2.5–27.3°
*b* = 8.2407 (10) Å	µ = 0.07 mm^−^^1^
*c* = 16.2671 (17) Å	*T* = 116 K
*V* = 1916.5 (4) Å^3^	Needle, red
*Z* = 4	0.70 × 0.20 × 0.10 mm

### Data collection

**Table:**

Bruker APEXII CCD diffractometer	1783 independent reflections
graphite	1764 reflections with *I* > 2σ(*I*)
Detector resolution: 8.333 pixels mm^-1^	*R*_int_ = 0.041
φ and ω scans	θ_max_ = 25.2°, θ_min_ = 2.5°
Absorption correction: multi-scan [Blessing (1995) and *SADABS* (Bruker, 2005)]	*h* = −17→17
*T*_min_ = 0.640, *T*_max_ = 0.746	*k* = −9→9
34258 measured reflections	*l* = −19→19

### Refinement

**Table:**

Refinement on *F*^2^	Primary atom site location: structure-invariant direct methods
Least-squares matrix: full	Secondary atom site location: difference Fourier map
*R*[*F*^2^ > 2σ(*F*^2^)] = 0.037	Hydrogen site location: inferred from neighbouring sites
*wR*(*F*^2^) = 0.086	H-atom parameters constrained
*S* = 1.23	*w* = 1/[σ^2^(*F*_o_^2^) + (0.0282*P*)^2^ + 0.7948*P*] where *P* = (*F*_o_^2^ + 2*F*_c_^2^)/3
1783 reflections	(Δ/σ)_max_ < 0.001
247 parameters	Δρ_max_ = 0.19 e Å^−^^3^
1 restraint	Δρ_min_ = −0.16 e Å^−^^3^

### Special details

**Table:**

Geometry. All e.s.d.'s (except the e.s.d. in the dihedral angle between two l.s. planes) are estimated using the full covariance matrix. The cell e.s.d.'s are taken into account individually in the estimation of e.s.d.'s in distances, angles and torsion angles; correlations between e.s.d.'s in cell parameters are only used when they are defined by crystal symmetry. An approximate (isotropic) treatment of cell e.s.d.'s is used for estimating e.s.d.'s involving l.s. planes.
Refinement. Refinement of *F*^2^ against ALL reflections. The weighted *R*-factor *wR* and goodness of fit *S* are based on *F*^2^, conventional *R*-factors *R* are based on *F*, with *F* set to zero for negative *F*^2^. The threshold expression of *F*^2^ > σ(*F*^2^) is used only for calculating *R*-factors(gt) *etc*. and is not relevant to the choice of reflections for refinement. *R*-factors based on *F*^2^ are statistically about twice as large as those based on *F*, and *R*- factors based on ALL data will be even larger.

### Fractional atomic coordinates and isotropic or equivalent isotropic displacement parameters (Å^2^)

**Table:**

	*x*	*y*	*z*	*U*_iso_*/*U*_eq_	
N1	0.78152 (16)	1.0321 (3)	0.45002 (13)	0.0248 (5)	
N2	0.64108 (16)	0.6649 (3)	0.43989 (14)	0.0238 (5)	
N3	0.58268 (16)	0.6112 (3)	0.38682 (14)	0.0261 (5)	
C1	0.7761 (2)	1.1095 (4)	0.36962 (19)	0.0333 (7)	
H1A	0.7164	1.1669	0.3645	0.050*	
H1B	0.7806	1.0267	0.3266	0.050*	
H1C	0.8277	1.1869	0.3637	0.050*	
C2	0.73892 (18)	0.8894 (3)	0.47008 (16)	0.0216 (6)	
C3	0.76949 (18)	0.8406 (3)	0.55730 (17)	0.0235 (6)	
C4	0.83106 (18)	0.9831 (3)	0.58035 (17)	0.0250 (6)	
C5	0.8798 (2)	1.0170 (4)	0.6516 (2)	0.0376 (7)	
H5	0.8786	0.9433	0.6965	0.045*	
C6	0.9309 (2)	1.1616 (4)	0.6568 (2)	0.0444 (9)	
H6	0.9645	1.1863	0.7056	0.053*	
C7	0.9329 (2)	1.2686 (4)	0.5914 (2)	0.0421 (8)	
H7	0.9679	1.3663	0.5962	0.050*	
C8	0.8851 (2)	1.2369 (4)	0.5189 (2)	0.0349 (7)	
H8	0.8866	1.3101	0.4738	0.042*	
C9	0.83496 (19)	1.0929 (3)	0.51568 (18)	0.0258 (6)	
C10	0.6853 (2)	0.8259 (4)	0.61544 (18)	0.0321 (7)	
H10A	0.6504	0.9283	0.6157	0.048*	
H10B	0.7073	0.8019	0.6712	0.048*	
H10C	0.6443	0.7381	0.5965	0.048*	
C11	0.8248 (2)	0.6797 (3)	0.55651 (19)	0.0276 (6)	
H11A	0.8479	0.6563	0.6120	0.041*	
H11B	0.8778	0.6891	0.5187	0.041*	
H11C	0.7837	0.5914	0.5384	0.041*	
C12	0.67785 (19)	0.8119 (3)	0.41935 (17)	0.0245 (6)	
H12	0.6606	0.8612	0.3688	0.029*	
C13	0.55193 (18)	0.4505 (3)	0.40219 (17)	0.0240 (6)	
C14	0.5934 (2)	0.3493 (4)	0.46096 (19)	0.0312 (7)	
H14	0.6403	0.3914	0.4964	0.037*	
C15	0.5666 (2)	0.1883 (3)	0.46788 (19)	0.0316 (7)	
H15	0.5948	0.1210	0.5083	0.038*	
C16	0.4991 (2)	0.1253 (3)	0.4164 (2)	0.0323 (7)	
H16	0.4833	0.0135	0.4191	0.039*	
C17	0.4546 (2)	0.2260 (3)	0.36077 (18)	0.0282 (6)	
H17	0.4065	0.1826	0.3271	0.034*	
C18	0.47830 (19)	0.3887 (3)	0.35274 (16)	0.0245 (6)	
C19	0.42423 (19)	0.4918 (3)	0.29410 (17)	0.0247 (6)	
C20	0.32655 (19)	0.4815 (3)	0.29415 (18)	0.0280 (6)	
H20	0.2962	0.4128	0.3327	0.034*	
C21	0.2735 (2)	0.5700 (4)	0.23883 (19)	0.0336 (7)	
H21	0.2073	0.5609	0.2397	0.040*	
C22	0.3156 (2)	0.6708 (4)	0.1826 (2)	0.0371 (7)	
H22	0.2789	0.7308	0.1446	0.045*	
C23	0.4127 (2)	0.6838 (4)	0.18199 (19)	0.0367 (8)	
H23	0.4424	0.7541	0.1438	0.044*	
C24	0.4663 (2)	0.5949 (4)	0.23681 (18)	0.0304 (7)	
H24	0.5325	0.6041	0.2354	0.036*	

### Atomic displacement parameters (Å^2^)

**Table:**

	*U*^11^	*U*^22^	*U*^33^	*U*^12^	*U*^13^	*U*^23^
N1	0.0280 (12)	0.0215 (11)	0.0248 (12)	0.0027 (9)	0.0003 (9)	0.0034 (10)
N2	0.0218 (11)	0.0250 (12)	0.0247 (11)	0.0029 (9)	−0.0005 (9)	−0.0013 (10)
N3	0.0282 (12)	0.0252 (12)	0.0248 (12)	0.0016 (10)	−0.0049 (10)	−0.0008 (10)
C1	0.0377 (16)	0.0289 (15)	0.0334 (16)	0.0005 (13)	0.0023 (13)	0.0120 (14)
C2	0.0218 (13)	0.0214 (12)	0.0216 (13)	0.0049 (11)	0.0021 (11)	0.0014 (12)
C3	0.0247 (14)	0.0243 (13)	0.0215 (13)	0.0042 (11)	−0.0006 (11)	0.0012 (12)
C4	0.0201 (13)	0.0302 (15)	0.0249 (13)	0.0072 (11)	0.0024 (11)	−0.0055 (12)
C5	0.0318 (16)	0.0471 (18)	0.0339 (16)	0.0055 (14)	−0.0057 (14)	−0.0054 (16)
C6	0.0334 (17)	0.056 (2)	0.0438 (19)	0.0024 (15)	−0.0076 (15)	−0.0196 (18)
C7	0.0269 (16)	0.0374 (18)	0.062 (2)	−0.0023 (14)	0.0000 (15)	−0.0175 (17)
C8	0.0273 (16)	0.0272 (15)	0.0502 (18)	0.0014 (12)	0.0067 (14)	−0.0066 (15)
C9	0.0210 (13)	0.0241 (14)	0.0324 (14)	0.0034 (11)	0.0036 (12)	−0.0065 (13)
C10	0.0287 (15)	0.0413 (18)	0.0264 (15)	0.0042 (14)	0.0048 (12)	0.0063 (13)
C11	0.0279 (14)	0.0273 (14)	0.0276 (14)	0.0031 (12)	−0.0016 (12)	0.0065 (13)
C12	0.0285 (14)	0.0228 (14)	0.0220 (13)	0.0030 (11)	−0.0004 (11)	0.0031 (11)
C13	0.0248 (14)	0.0238 (13)	0.0234 (13)	0.0049 (11)	0.0057 (11)	−0.0019 (11)
C14	0.0301 (15)	0.0330 (16)	0.0305 (15)	0.0051 (12)	0.0007 (13)	−0.0011 (13)
C15	0.0336 (16)	0.0267 (15)	0.0345 (16)	0.0106 (12)	0.0096 (13)	0.0099 (14)
C16	0.0336 (16)	0.0243 (14)	0.0391 (16)	0.0006 (13)	0.0152 (13)	0.0016 (14)
C17	0.0255 (14)	0.0287 (15)	0.0304 (15)	−0.0037 (12)	0.0087 (12)	−0.0015 (12)
C18	0.0254 (14)	0.0269 (14)	0.0211 (13)	0.0014 (11)	0.0088 (11)	−0.0003 (11)
C19	0.0286 (14)	0.0238 (13)	0.0217 (13)	−0.0018 (11)	0.0002 (12)	−0.0067 (12)
C20	0.0288 (15)	0.0272 (15)	0.0282 (14)	−0.0041 (12)	0.0000 (13)	−0.0076 (13)
C21	0.0303 (16)	0.0348 (16)	0.0356 (16)	0.0009 (13)	−0.0067 (13)	−0.0118 (14)
C22	0.0432 (18)	0.0377 (17)	0.0304 (16)	0.0063 (15)	−0.0137 (14)	−0.0030 (14)
C23	0.0448 (19)	0.0423 (19)	0.0230 (15)	−0.0037 (15)	−0.0044 (14)	0.0070 (14)
C24	0.0287 (15)	0.0367 (17)	0.0258 (14)	−0.0031 (13)	0.0032 (12)	−0.0005 (13)

### Geometric parameters (Å, °)

**Table:**

N1—C2	1.364 (3)	C11—H11A	0.9800
N1—C9	1.405 (4)	C11—H11B	0.9800
N1—C1	1.457 (4)	C11—H11C	0.9800
N2—N3	1.280 (3)	C12—H12	0.9500
N2—C12	1.362 (4)	C13—C14	1.400 (4)
N3—C13	1.418 (4)	C13—C18	1.419 (4)
C1—H1A	0.9800	C14—C15	1.386 (4)
C1—H1B	0.9800	C14—H14	0.9500
C1—H1C	0.9800	C15—C16	1.379 (5)
C2—C12	1.361 (4)	C15—H15	0.9500
C2—C3	1.538 (4)	C16—C17	1.383 (4)
C3—C4	1.515 (4)	C16—H16	0.9500
C3—C10	1.536 (4)	C17—C18	1.389 (4)
C3—C11	1.544 (4)	C17—H17	0.9500
C4—C5	1.381 (4)	C18—C19	1.493 (4)
C4—C9	1.388 (4)	C19—C24	1.397 (4)
C5—C6	1.401 (5)	C19—C20	1.399 (4)
C5—H5	0.9500	C20—C21	1.384 (4)
C6—C7	1.381 (5)	C20—H20	0.9500
C6—H6	0.9500	C21—C22	1.375 (5)
C7—C8	1.389 (5)	C21—H21	0.9500
C7—H7	0.9500	C22—C23	1.392 (5)
C8—C9	1.388 (4)	C22—H22	0.9500
C8—H8	0.9500	C23—C24	1.385 (4)
C10—H10A	0.9800	C23—H23	0.9500
C10—H10B	0.9800	C24—H24	0.9500
C10—H10C	0.9800		
			
C2—N1—C9	111.6 (2)	C3—C11—H11B	109.5
C2—N1—C1	124.6 (2)	H11A—C11—H11B	109.5
C9—N1—C1	123.7 (2)	C3—C11—H11C	109.5
N3—N2—C12	113.2 (2)	H11A—C11—H11C	109.5
N2—N3—C13	114.0 (2)	H11B—C11—H11C	109.5
N1—C1—H1A	109.5	C2—C12—N2	121.1 (2)
N1—C1—H1B	109.5	C2—C12—H12	119.5
H1A—C1—H1B	109.5	N2—C12—H12	119.5
N1—C1—H1C	109.5	C14—C13—N3	123.0 (3)
H1A—C1—H1C	109.5	C14—C13—C18	119.2 (3)
H1B—C1—H1C	109.5	N3—C13—C18	117.7 (2)
C12—C2—N1	123.1 (2)	C15—C14—C13	120.6 (3)
C12—C2—C3	128.3 (2)	C15—C14—H14	119.7
N1—C2—C3	108.6 (2)	C13—C14—H14	119.7
C4—C3—C10	111.4 (2)	C16—C15—C14	120.3 (3)
C4—C3—C2	101.0 (2)	C16—C15—H15	119.8
C10—C3—C2	111.5 (2)	C14—C15—H15	119.8
C4—C3—C11	111.8 (2)	C15—C16—C17	119.6 (3)
C10—C3—C11	109.8 (2)	C15—C16—H16	120.2
C2—C3—C11	111.3 (2)	C17—C16—H16	120.2
C5—C4—C9	119.0 (3)	C16—C17—C18	121.9 (3)
C5—C4—C3	131.1 (3)	C16—C17—H17	119.0
C9—C4—C3	109.9 (2)	C18—C17—H17	119.0
C4—C5—C6	119.0 (3)	C17—C18—C13	118.3 (3)
C4—C5—H5	120.5	C17—C18—C19	118.9 (3)
C6—C5—H5	120.5	C13—C18—C19	122.8 (2)
C7—C6—C5	120.5 (3)	C24—C19—C20	117.8 (3)
C7—C6—H6	119.7	C24—C19—C18	123.3 (2)
C5—C6—H6	119.7	C20—C19—C18	118.8 (3)
C6—C7—C8	121.6 (3)	C21—C20—C19	120.9 (3)
C6—C7—H7	119.2	C21—C20—H20	119.5
C8—C7—H7	119.2	C19—C20—H20	119.5
C9—C8—C7	116.6 (3)	C22—C21—C20	120.8 (3)
C9—C8—H8	121.7	C22—C21—H21	119.6
C7—C8—H8	121.7	C20—C21—H21	119.6
C8—C9—C4	123.3 (3)	C21—C22—C23	119.2 (3)
C8—C9—N1	127.9 (3)	C21—C22—H22	120.4
C4—C9—N1	108.8 (2)	C23—C22—H22	120.4
C3—C10—H10A	109.5	C24—C23—C22	120.4 (3)
C3—C10—H10B	109.5	C24—C23—H23	119.8
H10A—C10—H10B	109.5	C22—C23—H23	119.8
C3—C10—H10C	109.5	C23—C24—C19	120.9 (3)
H10A—C10—H10C	109.5	C23—C24—H24	119.6
H10B—C10—H10C	109.5	C19—C24—H24	119.6
C3—C11—H11A	109.5		
			
C12—N2—N3—C13	173.1 (2)	C2—N1—C9—C4	2.7 (3)
C9—N1—C2—C12	175.1 (2)	C1—N1—C9—C4	−174.8 (2)
C1—N1—C2—C12	−7.4 (4)	N1—C2—C12—N2	176.2 (2)
C9—N1—C2—C3	−3.1 (3)	C3—C2—C12—N2	−6.1 (4)
C1—N1—C2—C3	174.4 (2)	N3—N2—C12—C2	177.9 (2)
C12—C2—C3—C4	−175.8 (3)	N2—N3—C13—C14	−10.7 (4)
N1—C2—C3—C4	2.2 (3)	N2—N3—C13—C18	172.1 (2)
C12—C2—C3—C10	−57.5 (4)	N3—C13—C14—C15	−173.7 (3)
N1—C2—C3—C10	120.6 (3)	C18—C13—C14—C15	3.5 (4)
C12—C2—C3—C11	65.5 (3)	C13—C14—C15—C16	0.5 (4)
N1—C2—C3—C11	−116.5 (2)	C14—C15—C16—C17	−3.5 (4)
C10—C3—C4—C5	60.5 (4)	C15—C16—C17—C18	2.5 (4)
C2—C3—C4—C5	179.0 (3)	C16—C17—C18—C13	1.5 (4)
C11—C3—C4—C5	−62.7 (4)	C16—C17—C18—C19	−177.2 (2)
C10—C3—C4—C9	−119.1 (2)	C14—C13—C18—C17	−4.4 (4)
C2—C3—C4—C9	−0.6 (3)	N3—C13—C18—C17	172.9 (2)
C11—C3—C4—C9	117.7 (3)	C14—C13—C18—C19	174.2 (3)
C9—C4—C5—C6	0.5 (4)	N3—C13—C18—C19	−8.5 (4)
C3—C4—C5—C6	−179.0 (3)	C17—C18—C19—C24	−132.0 (3)
C4—C5—C6—C7	−0.2 (5)	C13—C18—C19—C24	49.4 (4)
C5—C6—C7—C8	−0.2 (5)	C17—C18—C19—C20	45.8 (4)
C6—C7—C8—C9	0.3 (4)	C13—C18—C19—C20	−132.8 (3)
C7—C8—C9—C4	0.1 (4)	C24—C19—C20—C21	0.4 (4)
C7—C8—C9—N1	−179.6 (3)	C18—C19—C20—C21	−177.5 (3)
C5—C4—C9—C8	−0.5 (4)	C19—C20—C21—C22	−0.2 (4)
C3—C4—C9—C8	179.2 (2)	C20—C21—C22—C23	−0.3 (5)
C5—C4—C9—N1	179.3 (2)	C21—C22—C23—C24	0.7 (5)
C3—C4—C9—N1	−1.1 (3)	C22—C23—C24—C19	−0.6 (5)
C2—N1—C9—C8	−177.6 (3)	C20—C19—C24—C23	0.0 (4)
C1—N1—C9—C8	4.9 (4)	C18—C19—C24—C23	177.8 (3)

### Hydrogen-bond geometry (Å, °)

**Table:**

Cg1, Cg2 and Cg3 are the centroids of the C19–C24, C4–C9 and C13–C18 rings, respectively.

**Table:**

*D*—H···*A*	*D*—H	H···*A*	*D*···*A*	*D*—H···*A*
C1—H1C···Cg1^i^	0.98	2.86	3.582 (3)	131
C1—H1A···Cg1^ii^	0.98	3.02	3.948 (3)	160
C11—H11B···Cg3^iii^	0.98	2.74	3.721 (3)	179
C22—H22···Cg2^iv^	0.95	2.76	3.645 (3)	155

Symmetry codes: (i) *x*+1/2, −*y*+2, *z*; (ii) *x*, *y*+1, *z*; (iii) *x*+1/2, −*y*+1, *z*; (iv) −*x*+1, −*y*+2, *z*−1/2.
